# Comparative Transcriptomic Analysis for Identification of Environmental-Responsive Genes in Seven Species of Threadfin Breams (*Nemipterus*)

**DOI:** 10.3390/ijms26157118

**Published:** 2025-07-23

**Authors:** Zhaoke Dang, Qiaer Wu, Yanbo Zhou, Liangming Wang, Yan Liu, Changping Yang, Manting Liu, Qijian Xie, Cheng Chen, Shengwei Ma, Binbin Shan

**Affiliations:** 1 College of Marine Living Resource Sciences and Management, Shanghai Ocean University, Shanghai 201306, China; dangzk1224@163.com; 2 South China Sea Fisheries Research Institute, Chinese Academy of Fisheries Sciences, Guangzhou 510300, China; wqe66@163.com (Q.W.); zhouyanbo@scsfri.ac.cn (Y.Z.); wangliangming@scsfri.ac.cn (L.W.); liuyan_0503@163.com (Y.L.); yangbing3524@163.com (C.Y.); tracie2022@163.com (M.L.); xieqijian2020@163.com (Q.X.); cchen077@163.com (C.C.); 3 Key Laboratory of Offshore Fisheries Development, Ministry of Agriculture and Rural Affairs, Guangzhou 510300, China; 4 Key Laboratory of Marine Ranching, Ministry of Agriculture Rural Affairs, Guangzhou 510300, China

**Keywords:** *Nemipterus*, RNA sequencing, comparative transcriptomics, environmental adaptability, phylogenetic relationships

## Abstract

Members of the genus *Nemipterus* are economically important fish species distributed in the tropical and subtropical Indo-West Pacific region. The majority of species in this genus inhabit waters with sandy–muddy substrates on the continental shelf, although different species are found at slightly varying water depths. In this study, we sequenced seven species within the genus *Nemipterus* after identifying the specimens using complementary morphological analysis and DNA barcoding. Each species yielded over 40,000,000 clean reads, totaling over 300,000,000 clean reads across the seven species. A total of 276,389 unigenes were obtained after de novo assembly and a total of 168,010 (60.79%) unigenes were annotated in the protein database. The comprehensive functional annotation based on the KOG, GO, and KEGG databases revealed that these unigenes are mainly associated with numerous physiological, metabolic, and molecular processes, and that the seven species exhibit similarity in these aspects. By constructing a phylogenetic tree and conducting divergence time analysis, we found that *N. bathybius* and *N. virgatus* diverged most recently, approximately during the Neogene Period (14.9 Mya). Compared with other species, *N. bathybius* and *N. virgatus* are distributed in deeper water layers. Therefore, we conducted selection pressure analysis using these two species as the foreground branches and identified several environmental-responsive genes. The results indicate that genes such as *aqp1*, *arrdc3*, ISP2, Hip, *ndufa1*, *ndufa3*, *pcyt1a*, *ctsk*, *col6a2*, *casp2* exhibit faster evolutionary rates during long-term adaptation to deep-water environments. Specifically, these genes are considered to be associated with adaptation to aquatic osmoregulation, temperature fluctuations, and skeletal development. This comprehensive analysis provides valuable insights into the evolutionary biology and environmental adaptability of threadfin breams, contributing to the conservation and sustainable management of these species.

## 1. Introduction

The family Nemipteridae holds significant importance within the order Perciformes. Currently, it is known worldwide that there are five genera within the family Nemipteridae, including *Nemipterus* (threadfin breams), *Pentapodus* (whiptail breams), *Scolopsis* (monocle bream), *Parascolopsis* (dwarf monocle bream) and *Scaevius* (coral breams) [1,2]. Among them, the genus *Nemipterus* is the largest genus, with approximately 26 species under it [3]. The morphological differences among closely related species of the genus *Nemipterus* are minimal, leading to many instances of synonyms and homonyms among the species [4,5,6]. Some of its species are important economic species in many countries including India [7], Japan, Indonesia, China [8] and others [9,10]. The genus are well known for their delicious taste and commercial importance. They are commonly called threadfin breams and many are targeted by fishermen [3,11,12].

The genus *Nemipterus* are a group of warm, small-to-moderate-sized benthic fishes primarily distributed in the tropical and subtropical Indo-West Pacific region (IWP), where they have diversified [13]. They primarily feed on crustaceans, cephalopods, or other small fish. Species of the genus *Nemipterus* occur on muddy and sandy bottoms of the continental shelf, with habitats typically at depths of 20–140 m [3,5]. However, *N. bathybius* has been observed at depths reaching up to 300 m [3], suggesting its capability to adapt to a broader range of depths. Due to the close phylogenetic relationships and similar ecological habits among species within the genus *Nemipterus*, as well as the overlap in their trophic niches [14,15], there may be potential interspecific competition. Environmental pressures often drive the evolution of species, including adjustments in key genes [16,17]. Most functions and gene expression are regulated by environmental factors such as temperature and osmotic pressure [18]. Therefore, these factors often determine the adaptability of fish in different environments. Previous studies have shown that physiological adaptations to environmental conditions drive niche differentiation during the adaptive evolution of teleost fish [19]. In addition to the impacts caused by environmental changes due to climate change, interspecific competition can also lead to changes in species distribution [20]. In-depth research on biological mechanisms has revealed that during the adaptive evolution of marine fish, their corresponding unique genes will undergo changes due to positive selection [21].

Currently, biological growth parameters of the *Nemipterus* [10,22,23,24,25] have been examined intensively. Furthermore, many previous studies have classified the species within the genus *Nemipterus* based on traditional morphological methods. However, due to the similar external morphology of the *Nemipterus* species and varying focuses of different studies, there are still many issues related to taxonomy and systematics development that remain to be resolved within this group [3,6]. With the advancement of biotechnology, the analysis of interspecific phylogeny within the genus *Nemipterus* has gradually shifted from morphological levels to molecular levels, with mitochondrial DNA markers being widely utilized. Specifically, this includes genes such as COI gene, Cyt b gene, and 16S rRNA gene, among others [14,26,27,28,29,30]. The genetic markers used in current research contain relatively limited genetic information. Therefore, there is a need for a technological approach that can obtain more comprehensive and in-depth genomic information, and thus, Next Generation Sequencing (NGS) technology has emerged.

Next generation sequencing (NGS) technology has the advantages of high throughput, high sensitivity, and high speed. The rapid development of NGS methods and technologies has made it possible to conduct large scale whole transcriptome sequencing projects [31,32]. RNA sequencing (RNA-seq) can evaluate absolute transcript levels of sequenced and its advantages are mainly reflected in: to begin with, RNA-Seq is highly sensitive; meanwhile, RNA-seq can accurately determine every single nucleotide of each transcript; then, RNA-Seq can conduct transcriptomic detection for any species without the design of a probe for known sequences [33,34]. RNA-seq technologies have been employed for studying both model and nonmodel organisms [35,36]. The transcriptome represents almost all effectively expressed genes in specific cells or organs. When genomic sequences are unavailable, transcriptome sequencing can provide a comprehensive understanding of the regulatory mechanisms involved in specific biological processes, based on the structures and functions of differentially expressed genes [37,38,39]. In addition, transcriptomic analysis enables simultaneous analyses of multiple processes, including protein homeostasis, metabolism and other regulatory cellular processes [40]. Current studies on the genus *Nemipterus* have primarily focused on mitochondrial genome analyses [41,42,43,44], with no transcriptomic investigations conducted to date.

This study conducted RNA-seq analysis on seven species within the genus *Nemipterus*, yielding a large amount of transcriptome data. A phylogenetic tree was constructed using COI sequences to explore the phylogenetic relationships among the seven species. Subsequently, comparative transcriptome analysis was used to identify a series of positively selected genes, ultimately identifying environmental-responsive genes. The results will provide data support for investigating the evolutionary relationships within the genus *Nemipterus* and will provide a certain research basis for discovering species evolution driven by environmental factors.

## 2. Results

### 2.1. Species Identification and Phylogenetic Inferences

**Figure 1:**
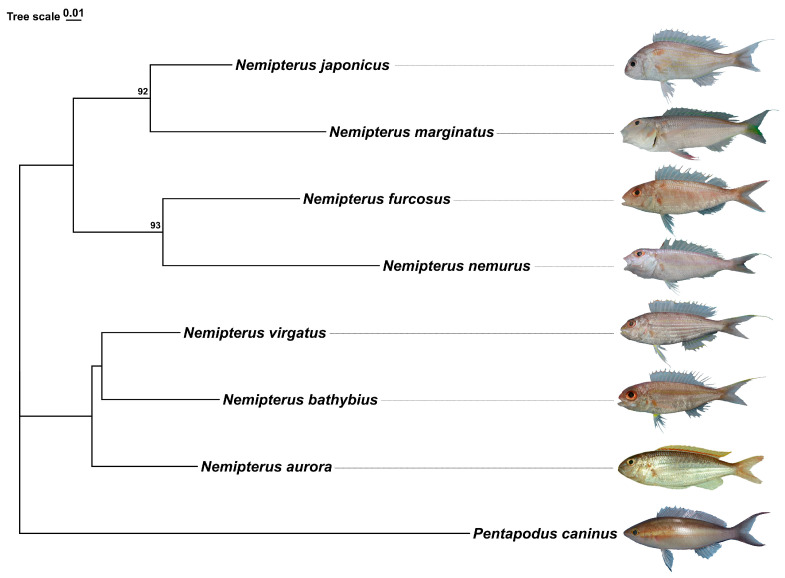
ML tree based on the analysis of COI sequences from seven species of *Nemipterus*. Note: Bootstrap values are shown near the nodes. The pictures in the phylogenetic tree were taken from samples collected in the Beibu Gulf, with the pictures of *N. aurora* were downloaded from Fishbase.

Morphological identification was used for seven species confirmation (*Nemipterus bathybius* Snyder, 1911, *N. furcosus* Valenciennes, 1830, *N. nemurus* Bleeker, 1857, *N. virgatus* Houttuyn, 1782, *N. aurora* Russell, 1993, *N. japonicus* Bloch, 1791, *N. marginatus*, Valenciennes, 1830). We amplified a 632 bp COI sequence from these seven species and the specific COI sequence is provided in the Supplementary Materials. Subsequently, we used *Pentapodus caninus* as an out-group and constructed a phylogenetic tree (Figure 1). In ML tree, seven species of *Nemipterus* are grouped into two clades. One of these clades includes *N. bathybius*, *N. virgatus* and *N. aurora* together. The remaining four species are gathered together. Among them, *N. marginatus* and *N. japonicus* appeared in a single clade; *N. nemurus* and *N. furcosus* also appeared in a single clade, with each clade having high bootstrap support (≥90%).

### 2.2. Transcriptome Sequencing and De Novo Assembly

Seven cDNA libraries were constructed and sequenced using Illumina novaseq 6000 by Gene Denovo Biotechnology Co. (Guangzhou, China). The sequencing information of transcriptomics is shown in Table 1—the seven species yielded a number of raw reads, ranging from 41,533,576 in *N. marginatus* to 55,724,924 in *N. furcosus*. In total, 345,064,922 reads were generated. After removing adapters, primers and low-quality reads, we obtained a total of 343,242,800 clean reads (99%) and the sequencing quality was high with Q20 ratio larger than 97% for all samples. These clean reads were subsequently assembled into transcripts and generated unigenes using the Trinity software (version 2.8.4). Finally, there were 32,661 unigenes in *N. bathybius*, 37,904 in *N. furcosus*, 38,342 in *N. nemurus*, 41,024 in *N. virgatus*, 34,882 in *N. aurora*, 40,036 in *N. japonicus* and 51,540 in *N. marginatus*. The summary of assembly analysis was displayed in Table 2.

The raw reads of the present study were uploaded to the SRA databases of NCBI under BioProject PRJNA1164947, with accession numbers SUB14748789.

### 2.3. Functional Annotation

The entire set of unigenes was annotated in four frequently used databases, including the NCBI non-redundant (Nr) protein, the Cluster of Orthologous Groups of proteins (KOG/COG) database, the SwissProt protein database, and the Kyoto Encyclopedia of Genes and Genomes (KEGG). The numbers and percentages of unigenes that matched to various databases for the seven species are shown in Table 3.

**Figure 2:**
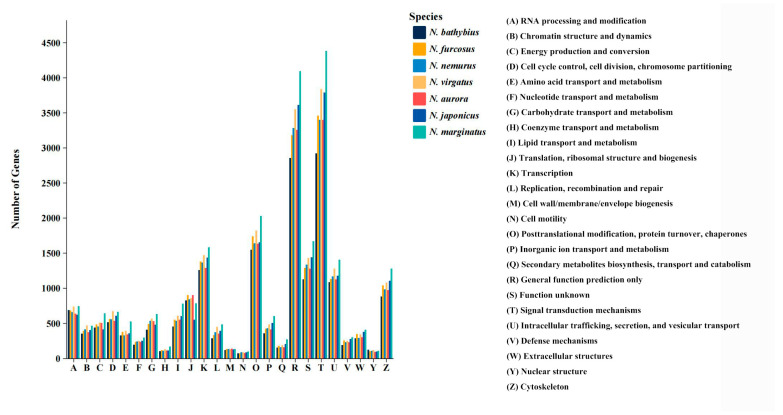
Histogram of the KOG annotations of unigenes in seven species. Note: The capital letters on the *x*-axis symbolize 25 biological processes, as shown on the right side and the *y*-axis represents the number of genes. Bars with different colors represent different species.

The unigenes of the seven species were annotated to the KOG database and were categorized into 25 subcategories (Figure 2). We found similar distribution patterns across the seven species. Among the 25 subcategories, “General function prediction only” (“R” term) and “Signal transduction mechanisms” (“T” term) are significantly higher than the other subcategories. Furthermore, some terms related to environmental adaptability are also enriched, such as “Posttranslational modification, protein turnover, chaperones” (“O” term) and “Intracellular trafficking, secretion, and vesicular transport” (“U” term).

**Figure 3:**
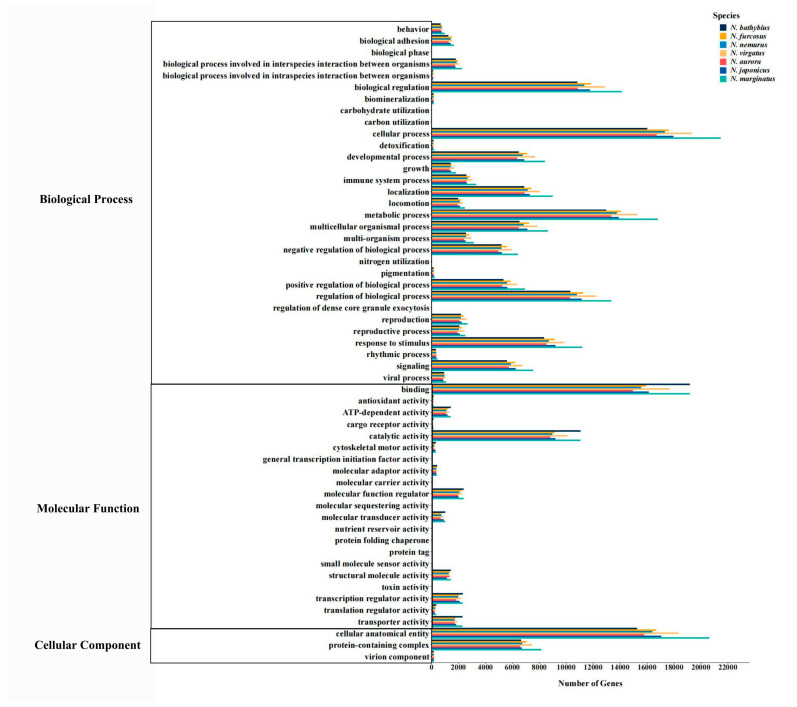
Histogram of the GO annotations of unigenes in seven species. Note: The *x*-axis represents GO terms number of genes and the *y*-axis represents GO terms belonging to three categories. Bars with different colors represent different species.

To determine the functional distribution of unigenes, we used the NR annotation information and obtained the GO annotation information of Unigenes with the Blast2GO software (version 2.6.0). All unigenes annotated in the GO database are divided into three major categories, including “biological processes”, “molecular functions”, and “cellular components” (Figure 3). Among them, “biological processes” contain the largest number of annotated unigenes, while “cellular components” has the fewest. Additionally, term “cellular process” dominates in the “biological process” category, term “binding” dominates in the “molecular function” category, and term “cellular anatomical entity” dominates in the “cellular component” category. These terms are all related to cellular composition and function, maintaining the basic regulation of the organism.

**Figure 4:**
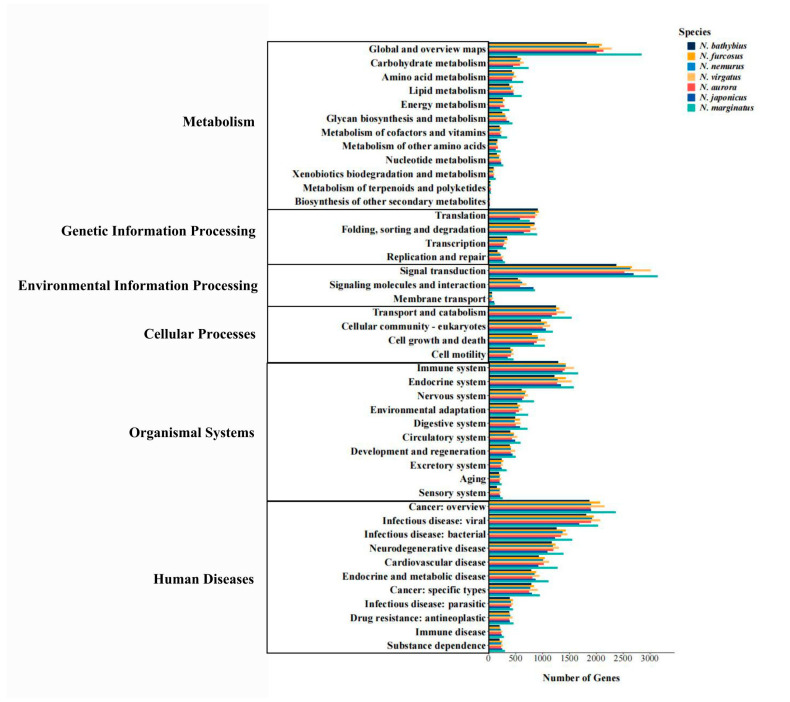
Histogram of the KEGG annotations of unigenes in seven species. Note: The *x*-axis represents the number of genes annotated to the KEGG pathways and the y-axis represents pathways in KEGG annotations. Bars with different colors represent different species.

To further investigate the complex behaviors and interactions of gene products in biology, we conducted KEGG pathway analysis. Six types of hierarchy one pathways were involved in the KEGG annotation in the seven species, including “Metabolism”, “Genetic Information Processing”, “Environmental Information Processing”, “Cellular Processes”, “Organismal Systems”, “Human Diseases” (Figure 4). Among them, the “Human Diseases” pathway contains the largest number of annotated sequences, followed by the “Organismal Systems” pathway. Furthermore, in hierarchy two pathways, most unigenes in the seven species were annotated in the “Global and overview maps”, “Signal transduction”, “Transport and catabolism”, “Immune system”, “Endocrine system”, “Cancer: overview”, and “Infectious disease: viral” pathways. These pathways are all involved in the transmission of information within organisms and their response to various internal and external environmental changes, thereby helping organisms maintain homeostasis and adaptability.

### 2.4. Functional Enrichment Analysis of Unique Genes

Using the combined method of Diamond and OrthoMCL, gene family information was established for seven species of the genus *Nemipterus* and *Pagrus major*. The analysis indicated that 4848 genes families were shared among the seven species, with 4546 gene families unique to species *N. bathybius*, 5178 gene families unique to species *N. furcosus*, 4085 gene families unique to species *N. nemurus*, 5280 gene families unique to species *N. virgatus*, 3891 gene families unique to species *N. aurora*, 5016 gene families unique to species *N. japonicus*, and 8316 gene families unique to species *N. marginatus*.

**Figure 5:**
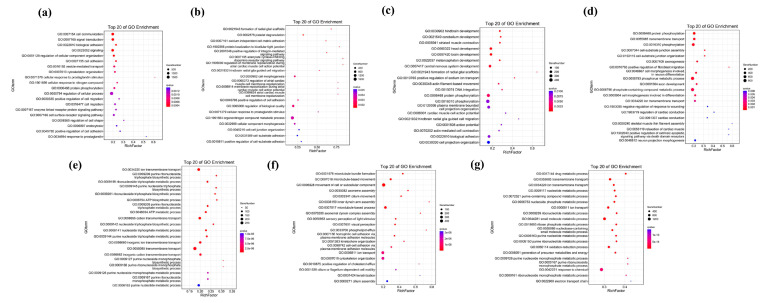
GO enrichment analysis of unique genes in seven species. Note: (**a**): *N. bathybius*; (**b**): *N. furcosus*; (**c**): *N. nemurus*; (**d**): *N. virgatus*; (**e**): *N. aurora*; (**f**): *N. japonicus*; (**g**): *N. marginatus*.

The GO enrichment analysis of unique gene families revealed that, within the biological processes, “cellular process” and “metabolic process” were the two most common GO terms. Meanwhile, ‘regulation of cellular process’ (GO:0050794) shows the highest level of enrichment in *N. bathybius*, ‘organonitrogen compound metabolic process’ (GO:1901564) in *N. furcosus*, ‘phosphorylation’ (GO:0016310) in *N. nemurus*, ‘phosphorus metabolic process’ (GO:0006793) in *N. virgatus*, ‘transmembrane transport’ (GO:0055085) in *N. aurora*, ‘ion transport’ (GO:0006811) in *N. japonicus*, and ‘response to chemical’ (GO:0042221) in *N. marginatus*. Within the molecular functions and cellular components, aside from *N. marginatus*, ‘carbohydrate derivative binding’ (GO:0097367) and ‘cytoskeleton’ (GO:0005856) demonstrate significant enrichment across all other species. In addition, the unique genes of *N. bathybius* are predominantly associated with ‘protein binding’ (GO:0005515) and ‘endomembrane system’ (GO:0012505), yet they do not exhibit significant enrichment in other species (Figure 5).

**Figure 6:**
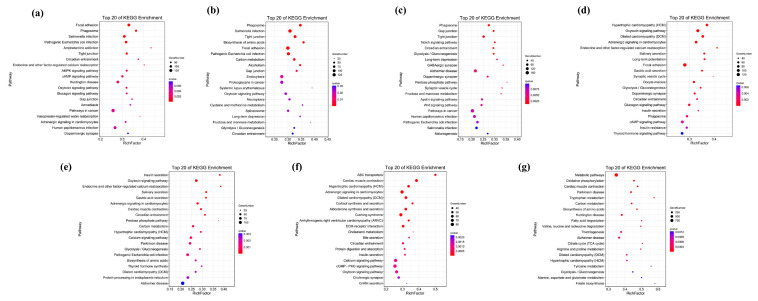
KEGG enrichment analysis of unique genes in seven species. Note: (**a**): *N. bathybius*; (**b**): *N. furcosus*; (**c**): *N. nemurus*; (**d**): *N. virgatus*; (**e**): *N. aurora*; (**f**): *N. japonicus*; (**g**): *N. marginatus*.

According to the KEGG pathway enrichment results (Figure 6), ‘Focal adhesion’ (ko04510) and ‘Alzheimer disease’(ko05010) are highly enriched in seven species. Among them, the unique genes of *N. marginatus* are mainly enriched in ‘Metabolic Pathways’ (ko01100), and the unique genes of *N. bathybius* are closely related to the ‘PI3K-Akt signaling pathway’ (ko04151).

### 2.5. Phylogenetic and Divergence Time Estimations

**Figure 7:**
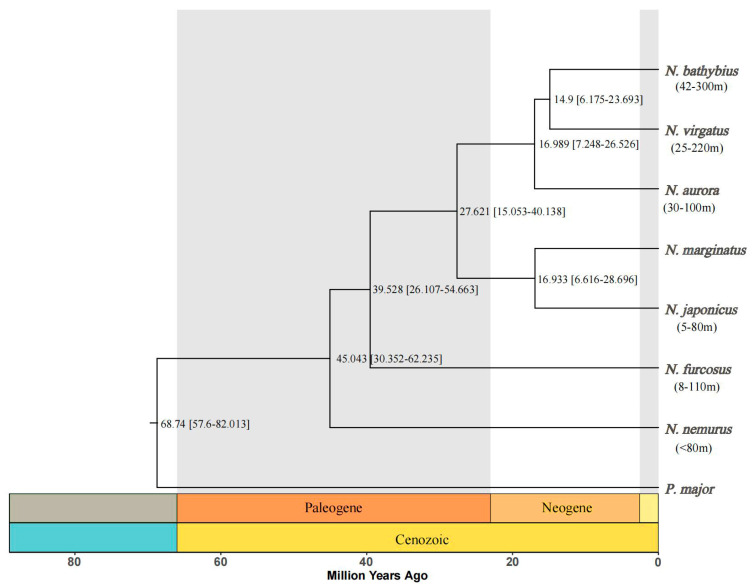
Phylogenetic tree and divergence times estimated of seven species of *Nemipterus* and *P. major*. Note: The content within parentheses under the species names represents the depth range of distribution for each species.

Phylogenetic and divergence time estimations analysis is based on single-copy orthologous genes. The evolutionary tree provides a hierarchical or topological representation, which reflects the divergence points of novel gene duplications or organisms sharing a common ancestor. The lengths of the branches indicate the evolutionary distance between the proteins that existed at the time of these events and the contemporary proteins (Figure 7). We amplified a 632 bp COI sequence from these seven species of the genus *Nemipterus*. The phylogenetic trees constructed from the COI barcode sequences of seven species of the genus *Nemipterus* in this study shows multiple clades. Specifically, *N. japonicus* and *N. marginatus* formed a clade, *N. furcosus* and *N. nemurus* grouped into another clade, while the remaining species—*N. bathybius*, *N. virgatus*, and *N. aurora*—clustered together to form a separate, independent clade. It is noteworthy that *N. bathybius* and *N. virgatus* is at the tip of the evolutionary branch. Meanwhile, we found that the divergence time estimation for seven species of the genus *Nemipterus* and *P. major* is 68 million years ago (Mya). The divergence events among the *Nemipterus* species predominantly occurred during the Cenozoic era, from the Paleogene to the Neogene epochs, spanning approximately 45 to 14.9 million years ago (Mya). Among the studied species, *N. nemurus* was the first to diverge at approximately 45 Mya, followed by *N. furcosus*. The remaining five species diverged predominantly during the Neogene period (27.6–14.9 Mya).

### 2.6. Selection Pressure Analysis

**Figure 8:**
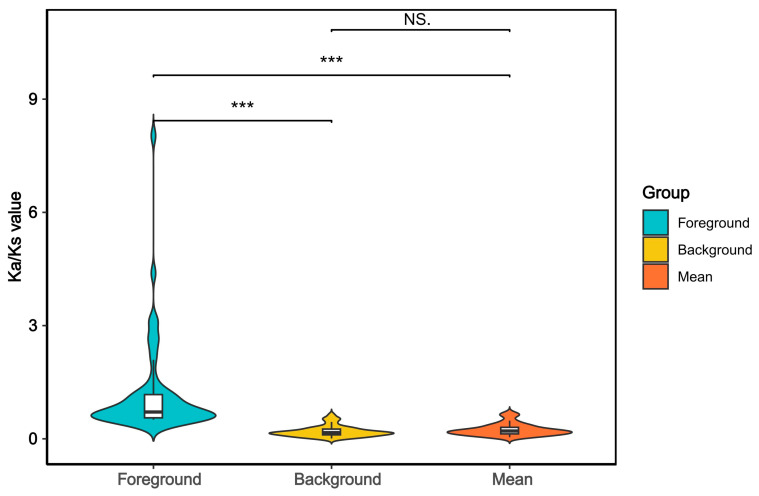
Violin plot comparing the Ka/Ks ratios across three groups (*p <* 0.05). Note: The *x*-axis represents different groups and the *y*-axis represents the Ka/Ks values. In the figure, asterisks “***” denote statistical significance with a *p*-value less than 0.001, whereas “NS.” signifies “Not Significant” indicating no statistically significant difference.

**Figure 9:**
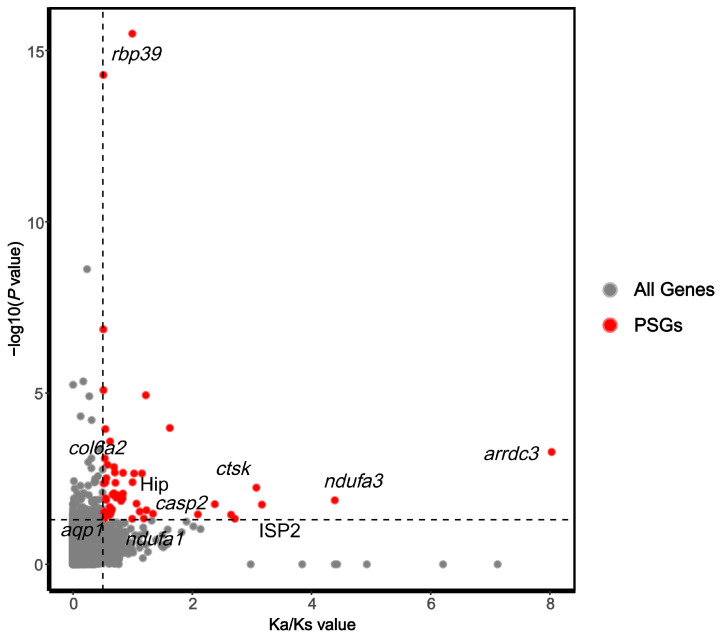
Volcano plot based on the foreground branch Ka/Ks values analysis. Note: The *x*-axis represents the Ka/Ks value and the *y*-axis represents the −log10-transformed *p* value. The red dots indicate positively selected genes. The horizontal dashed line corresponds to a *p*-value of 0.05, and the vertical dashed line indicates a Ka/Ks value of 0.5.

Conduct selection pressure analysis based on single-copy orthologous genes to identify genes under selection during evolution. Given that the maximum habitat depths of *N. bathybius* and *N. virgatus* reach 300 m and 220 m, respectively [5], and our phylogenetic analysis places *N. bathybius* and *N. virgatus* at the terminal positions of the clade, we designated these two species as foreground branches and the remaining species as the background branches to detect genes under positive selection linked to depth adaptation. We conducted selection pressure analysis by comparing the differences in the Ka/Ks radios at the gene level [45]. Genes with *p* values < 0.05 are considered to evolve more rapidly. We calculated the Ka/Ks values for the foreground branches, background branches, and all species, and compared the differences among the three groups (*p* < 0.05) (Figure 8). The results show that the Ka/Ks values of the foreground branches is significantly higher, while the background branches have the lowest value. After removing outliers, a total of 52 genes representing positive selection were identified (Table S1). Significance analysis of positively selected genes based on the foreground branch Ka/Ks values (Figure 9). These genes are involved in various functions such as signal transduction, cell apoptosis, metabolic processes, immune response, and membrane transport. We identified several environmental-responsive genes, such as *aqp1*, *arrdc3*, ISP2, Hip, *ndufa1*, *ndufa3*, *pcyt1a*, *ctsk*, *col6a2*, *casp2*. Among these, we selected genes that are under strong positive selection and calculated their genetic distances among seven species, constructing Neighbor-Joining (NJ) trees (Table S2, Figures S1 and S2).

## 3. Discussion

This study primarily investigates the phylogenetic relationships within the genus *Nemipterus* and explores the potential molecular pathways and genes that facilitate adaptation to distinct ecological niches among species. We first utilized DNA barcoding as a molecular approach to authenticate the taxonomic identity of the sampled specimens. Subsequently, we utilized RNA from seven species of the genus *Nemipterus* and applied Illumina sequencing technology for transcriptome de novo assembly and functional annotation. The phylogenetic relationships among species within the genus *Nemipterus* were explored by constructing a phylogenetic tree, and then environmental-responsive genes were identified through comparative transcriptome analysis.

### 3.1. Transcriptomic Characteristics of the Genus Nemipterus

This study used RNA-seq technology to obtain transcriptome information for seven species of the genus *Nemipterus*. Each species yielded over 40,000,000 clean reads, totaling over 300,000,000 clean reads across the seven species. The Q30 scores of the seven species ranged from 92.37% to 94.45%, indicating high sequencing quality. A total of 276,389 unigenes were obtained after assembly and a total of 168,010 (60.79%) unigenes were annotated in the protein database. Among them, the unigene annotation rate of *N. aurora* (64.02%) is higher than that of other species, which may be due to its higher N50 value (1958 bp).

Based on the KOG classification of proteins, more than 36% of the transcripts in all seven species were classified into 25 functional categories. In particular, the categories of “General function prediction only” and “Signal transduction mechanisms” showed significant dominance and were much higher than the other categories. The dominance of these two categories highlights the active regulation of the relationship between cells and the external environment in species of the genus *Nemipterus*, as well as the significant importance in the exploration of unknown gene functions. In the level two GO categories of biological process and cellular component, most of the unigenes were involved in maintaining basic biological functions, such as “cellular process”, “metabolic process” and “cellular anatomical entity”. In terms of molecular function, the majority of unigenes were primarily involved in “binding” and “catalytic activity” processes, reflecting their active role in maintaining fundamental life processes. For KEGG pathways, they were mainly annotated in the following pathways: “Global and overview maps”, “Signal transduction”, “Transport and catabolism”, “Immune system”, “Endocrine system”, “Cancer: overview” and “Infectious disease: viral”. The annotation results show that the seven species exhibit similar pathway distributions, indicating that they have a high degree of conservation in basic metabolic pathways and cellular functions [46]. The transcriptomic dataset obtained from this study on seven species within the genus *Nemipterus*, economically important fish in many countries, addresses a knowledge gap and enables further in-depth research into the phylogeny and adaptive evolution of the genus *Nemipterus*.

### 3.2. Phylogenetic and Divergence Time Analysis of the Genus Nemipterus

The COI gene, with its moderate evolutionary rate, is widely recognized as an effective tool for molecular species identification and resolving phylogenetic relationships among closely related species [47,48]. In this study, the phylogenetic tree constructed based on the COI barcode sequences of the seven species of the genus *Nemipterus* revealed multiple clades. The present findings are consistent with previously observed topological structures in certain studies. For example, *N. japonicus* and *N. marginatus* clustered into a single clade, indicating their close phylogenetic relationship [13,14]. Compared to other species within the genus *Nemipterus*, *N. bathybius* exhibited closer affinity to *N. virgatus* [14,49], while *N. furcosus* and *N. nemurus* formed a distinct sister group [13]. However, discrepancies exist with some studies proposing a closer relationship between *N. japonicus* and *N. nemurus* [30], which conflicts with the topology resolved in this study. Overall, these results demonstrate that the COI gene effectively elucidates taxonomic relationships among *Nemipterus* species.

A total of 2923 orthologous gene families were identified across seven species, and subsequently conducted divergence time analysis based on single-copy orthologous genes. We found that species within the *Nemipterus* genus diverged predominantly during the Neogene period, aligning with the hypothesis that the Neogene represents a critical phase for marine fish speciation [50,51]. Concurrently, the Cenozoic era was marked by profound and complex climatic transformations [52], with studies indicating that Neogene temperature fluctuations were pivotal in shaping modern marine biodiversity patterns [53]. The rapid diversification observed in the genus *Nemipterus* during this period likely reflects adaptive responses to climatic and environmental shifts. Similar divergence timelines have been documented in other marine taxa [54,55,56], further underscoring the Neogene as a key epoch for evolutionary radiation in marine ecosystems. Notably, *N. bathybius* is the most recent species on the evolutionary tree, with a divergence time of approximately 14.9 Mya. Due to *N. bathybius* having the ability to adapt to a wider range of depths, the phylogenetic analysis may suggest an evolutionary transition from shallow- to deep-water species within the genus *Nemipterus*. The ability to live across wide depths may promote population connectivity [57]. The increase in habitat depth may have advantages such as improved environmental stability and reduced metabolic energy consumption [58]. Previous studies have shown that organisms inhabiting deeper water layers need to functionally adapt to the prevalent high-pressure and low-temperature conditions [59].

### 3.3. Adaptive Evolution of the Genus Nemipterus

Environmental stress perturbs cellular homeostasis, thereby impairing critical physiological processes at the organismal level [60]. Responses to environmental stress generally range from neuroendocrine and hormone release to changes in metabolism, cellular function, and circulation, and finally to overall physiological and behavioral stress responses [61]. Marine organisms undergo a series of physiological, biochemical, and molecular activities to cope with changes in environmental stress [62,63]. For *N. bathybius* and *N. virgatus*, which have broader bathymetric ranges, they may encounter gradients in salinity, water temperature, and light intensity during vertical migration into deeper waters [64,65]. Marine teleosts must ingest the seawater medium to replace the water loss osmotically, and the molecular mechanisms underlying this process are of vital importance [66]. In this study, we identified numerous genes involved in signal transduction and cellular regulation, with a particular focus on those related to environmental adaptation. Among these genes, *aqp1* participates in osmoregulatory pathways. Pairwise interspecific sequence divergence analyses revealed genetic differentiation in *N. marginatus* relative to congeners, suggesting that *aqp1* may have undergone lineage-specific adaptive divergence during the evolutionary history of this taxon. This finding aligns with the osmotic demands imposed by the fluctuating salinity of estuarine and coastal habitats occupied by this species [67], implying that the adaptive evolution of *aqp1* constitutes a key mechanism for coping with such osmotic environments. Aquaporins, AQPs, are members of a ubiquitous family of channel-forming proteins [68]. AQP1 originally characterized in human erythrocytes, represents a water-selective channel protein [69]. The induction of *aqp1* gene by hypertonicity results from a sequential MAPK-signaling pathway that directly affects the promoter region of *aqp1* gene [70]. Aquaporin proteins can regulate the water permeability of cell membranes to maintain cell volume and counteract the osmotic imbalance caused by the hydrostatic pressure of the deep sea. In teleost fish, aquaporins have been demonstrated to potentially play multiple roles in osmoregulatory processes [71]. In addition to its role in osmoregulation, *aqp1* has been identified in photoreceptor cells of the mouse retina [72]. Furthermore, it has been found in the retinal pigment epithelium (RPE), where it is associated with transepithelial water transport [73]. It can be hypothesized that the *aqp1* gene may have undergone positive selection in the threadfin breams to adapt to low-light conditions. Notably, AQP4, a paralog within the aquaporin family, has been functionally validated as essential for maintaining retinal signal transduction [74]. Additionally, significant positive selection signals were detected on the foreground branch (Ka/Ks = 8, *p* < 0.001), suggesting *arrdc3* underwent accelerated adaptive evolution in this lineage, potentially driven by osmoregulatory challenges. Arrestin domain containing protein 3 is a member of the α-arrestin family [75] and can influence energy balance by regulating thermogenic function in adipose tissue [76]. *arrdc3* has been identified to exhibit positive selection signals in the euryhaline fish *Acanthopagrus latus*, potentially associated with salinity adaptation [77]. Furthermore, this gene has been implicated in osmoregulatory processes in *Scophthalmus maximus* [78]. Based on the genetic distance matrix, significant differences in genetic distances among seven species for the *arrdc3* gene are observed. Specifically, *N. bathybius* exhibits relatively lower divergence with *N. virgatus* and *N. aurora*, while showing generally higher genetic differences with other species. This pattern aligns with the phylogenetic affiliations as determined by their evolutionary history. The smallest genetic divergence between *N. japonicus* and *N. marglnatus* (0.0258) suggests that the function of the *arrdc3* gene may be relatively conserved within this small evolutionary clade.

Fluctuations in water temperature critically impair the physiological functions of aquatic organisms, prompting teleosts to dynamically modulate physiological, biochemical, and molecular processes as adaptive strategies to thermal variations [60]. In this study, we also identified proteins with positive selection signals associated with temperature adaptation, such as Hip (Ka/Ks > 1, *p* < 0.05) and ISP2 (Ka/Ks > 3, *p* < 0.05). Heat shock proteins (HSPs) also called molecular chaperones, are expressed in response to heat or other forms of stress [79]. Hsp70 interacting protein (Hip) is a co-factor (co-chaperone) of the 70 kDa heat shock proteins (Hsc/Hsp70) [80]. The HSP70 gene family has been detected and identified in various species of teleost fish and is closely related to environmental responses, such as heat, hypoxia, and salinity stress [81,82,83]. Hip also has been associated with salinity acclimation/adaptation in teleosts [84]. *st13* encodes the Hip protein, and genetic distance results indicate that the *st13* gene exhibits an overall conserved pattern of genetic differentiation across seven species, with all pairwise genetic distances remaining at relatively low levels (0.0224–0.0697). Ice structure proteins (ISPs), also known as antifreeze proteins, can protect organisms from freezing temperatures [85]. AFPs enhance the ability of fish to resist freezing at low temperatures [86]. Studies have demonstrated that the large yellow croaker can synthesize ISP2 protein to protect the central neural system from cold stress [87]. Additionally, the genes *ndufa1*, *ndufa3* and *pcyt1a* were also found to exhibit positive selection (Ka/Ks > 1). The genes *ndufa3* and *ndufa1* have been identified as potentially related to the adaptation of the threadfin breams to the low-temperature environment of the deep sea. In some teleost fish, the ability to synthesize ATP through oxidative phosphorylation is enhanced at lower temperatures [60]. The expression of *ndufa3* also can facilitate adaptive metabolic adjustments in response to hypoxic environments in organisms [88]. The *pcyt1a* gene encodes phosphocholine cytidylyltransferase α (CCTα), which is considered to be involved in the lipid metabolism process of teleost fish [89]. Based on our findings, genus *Nemipterus* may modulate energy metabolism to adapt to temperature variations in its environment.

There are significant differences in physical environments between deep and shallow water, including water pressure. For aquatic organisms, the skeleton can adjust its structure to adapt to physiological stimuli or mechanical stress. The adaptation mechanisms involve multiple cellular signaling processes [90]. We have identified the *ctsk* gene as being under strong positive selection (Ka/Ks > 3) and this gene also exhibits low genetic distances among species pairs (0.00707–0.06364), indicating that its amino acid sequence may play a crucial fundamental biological role in the evolution within the *Nemipterus* genus. Additionally, the clustering of *N. bathybius*, *N. virgatus*, and *N. aurora* into one group, and *N. japonicus* and *N. marginatus* into another, further substantiates the reliability of the phylogenetic relationships within this genus. Cathepsin K (*ctsk*) is a cysteine protease predominantly produced by osteoclasts. It can mediate bone degradation and regulate skeletal growth [91]. A reduction in bone mass can help decrease body weight, which may enhance locomotor ability and swimming speed, as well as enable them to withstand the immense pressure of the deep sea [92]. Two additional positively selected genes associated with skeletal development are *col6a2* and *casp2*. The *col6a2* gene encodes the α2 chain of collagen type VI, which is an essential component of collagen type VI [93]. Collagen type VI plays a crucial role in maintaining the structural integrity and development of bones and soft tissues [94]. Previous studies have demonstrated that caspase-2 can also be directly involved in skeletal muscle differentiation and myogenesis [95]. The discovery of these genes reflects the molecular mechanisms by which the genus *Nemipterus* adapts to the high-pressure deep-sea environment through its skeletal system. In summary, our study demonstrates that the adaptation of the threadfin breams to environmental changes involves the regulation of numerous genes and proteins. The identification of these candidate genes provides a reference for further understanding the mechanisms driving the evolution of species within the genus *Nemipterus*.

## 4. Material and Methods

### 4.1. Sample Collection and Species Identification

All samples were collected from the South China Sea using a bottom trawl method, and the collected samples were identified to species where possible using morphological keys in taxonomic references. The morphological identification was primarily based on FAO catalog of genus *Nemipterus* [3] and Systematic Synopsis of Chinese Fishes [96] and then checked against photographs available on FishBase (http://www.fishbase.org; accessed on 18 October 2024). Furthermore, we employed DNA barcoding to confirm the species identification by comparing the sequences with those in GenBank. We used primers FishF1 (5′-TCA ACC AAC CAC AAA GAC ATT GGC AC-3′) and FishR1 (5′-TAG ACT TCT GGG TGG CCA AAG AAT CA-3′) to amplify the mitochondrial COI gene by PCR [48]. Then, we used *Pentapodus caninus* as an outgroup and constructed a maximum likelihood (ML) tree using IQ-Tree2 (version 2.2.2.6) (https://github.com/StaPH-B/docker-builds/pull/679; accessed on 20 October 2024).

One individual was collected for each species, specimens and environment information can be found in Table S3. These samples were sacrificed after anaesthetization with MS-222 (100 mg/L). Dorsal muscle tissue was sampled, frozen immediately in liquid nitrogen, and stored at −80 °C for subsequent experiments. Animal handling during sample collection complied with institutional standards (see Institutional Review Board Statement for full details).

### 4.2. RNA Extraction, cDNA Library Building and Illumina Sequencing

We collected muscle tissue samples from one individual of each species for RNA extraction. Total RNA was extracted using Trizol reagent kit (Invitrogen, Carlsbad, CA, USA) according to the manufacturer’s protocol. RNA degradation and contamination was assessed with 1% agarose gels. The RNA integrity was assessed on an Agilent 2100 Bioanalyzer (Agilent Technologies, Palo Alto, CA, USA). And the samples with RNA Integrity Number (RIN) ≥ 7 indicate that the integrity of the extracted RNA is sufficient for subsequent transcriptome sequencing requirements [97].

After total RNA was extracted, eukaryotic mRNA was enriched by Oligo(dT) beads, while prokaryotic mRNA was enriched by removing rRNA by Ribo-Zero^TM^ Magnetic Kit (Epicentre, Madison, WI, USA). Then, the enriched mRNA was fragmented into short fragments using fragmentation buffer and reverse transcribed into cDNA with random primers. Second-strand cDNA was synthesized by DNA polymerase I, RNase H, dNTP and buffer. Then, the cDNA fragments were purified with QiaQuick PCR extraction kit (Qiagen, Venlo, The Netherlands), end repaired, a base added, and ligated to Illumina sequencing adapters. The ligation products were size selected by agarose gel electrophoresis, PCR amplified, and sequenced using Illumina novaseq 6000 by Gene Denovo Biotechnology Co. (Guangzhou, China).

### 4.3. Transcriptome De Novo Assembly and Functional Annotation

The reads were filtered using fastp [98] (version 0.18.0) and clean reads were obtained by removing raw reads containing adapter, ploy-N (N ratio > 10%) and low quality reads containing more than 50% of low quality (*Q*-value ≤ 20) bases. Transcriptome de novo assembly was carried out with short reads assembling program-Trinity [99] (version 2.8.4), with the min_kmer_cov set to 2. The k-mer value was set to 25. Subsequently, we assessed the completeness of the assembly information using BUSCO software (version 3.0). To annotate the unigenes, we used BLASTx program (http://www.ncbi.nlm.nih.gov/BLAST/; accessed on 22 October 2024) with an E-value threshold of 1 × 10^−5^ to NCBI non-redundant protein (Nr) database (http://www.ncbi.nlm.nih.gov; accessed on 22 October 2024), the Swiss-Prot protein database (http://www.expasy.ch/sprot; accessed on 22 October 2024), the Kyoto Encyclopedia of Genes and Genomes (KEGG) database (http://www.genome.jp/kegg; accessed on 22 October 2024), and the COG/KOG database (http://www.ncbi.nlm.nih.gov/COG; accessed on 22 October 2024). Protein functional annotations could then be obtained according to the best alignment results.

### 4.4. Differential Gene Expression Analysis

We used the software package RSEM [100] (version 1.2.19) to estimate the gene expression levels for each species. First, read_count values of all genes were obtained by mapping clean data back onto the transcripts. Then, we calculated the TPM (Transcripts Per Kilobase of exon model per Million mapped reads) values. RNAs differential expression analysis was performed by DESeq2 (version 3.22) [101] software. The genes with the parameter of false discovery rate (FDR) below 0.05 and absolute fold change ≥ 2 were considered differentially expressed genes. We selected *Pagrus major* as the outgroup and used the default settings of the Diamond (version 2.1.12) [102] and OrthoMCL (version 2.0) [103] software to identify orthologous unigenes among species. After that the orthologous genes between species were classified into one protein family, other species specific genes were also classified into different families by OrthoMCL v2.0. The KEGG and GO function annotation and enrichment analysis of obtained specific genes of each species were then conducted. Following multiple testing correction, pathways with a *Q*-value of ≤0.05 are delineated as significantly enriched among differentially expressed proteins.

### 4.5. Single Copy Orthologous Gene Analysis

In gene family research, most studies are based on single-copy orthologous genes. Initially, multiple sequence alignment was performed on distinct protein sequences within the same single-copy orthologous gene family using MUSCLE (version 5.0) [104] software (http://www.drive5.com/muscle/; accessed on 24 October 2024). After sequence alignment, the evolutionary tree was constructed using NJ and ML methods. The NJ tree was constructed with MEGA [105], and the ML tree was constructed with iqtree [106]. Then, the Bootstrap method was used to test 1000 times to construct the final evolution trees. Estimating species divergence is conducted using the mcmctree (http://abacus.gene.ucl.ac.uk/software/paml.html; accessed on 24 October 2024) tool in the PAML [107] package. Given the considerable variation in depth distribution ranges among species within the genus *Nemipterus*, particularly the documented presence of *N. bathybius* and *N. virgatus* at depths exceeding 200 m [3,5]. Therefore, comparative transcriptomics was utilized and selection pressure analysis was conducted by calculating Ka, Ks, and the Ka/Ks ratio using paml-codeml. Positively selected genes exhibiting strong signatures were subsequently subjected to pairwise interspecific sequence divergence analysis in MEGA to explore the molecular mechanisms underlying the adaptation of this genus to deep-sea environments.

## 5. Conclusions

This study conducted de novo assembly and sequencing of the transcriptomes of seven species within the genus *Nemipterus*, enriching the genomic information of the genus. Furthermore, comparative transcriptome analysis was used to elucidate the phylogeny and estimate the divergence times of the seven species. Additionally, through Ka/Ks analysis, a number of genes responsive to environmental changes were identified. The results indicate that genes such as *aqp1*, *arrdc3*, ISP2, Hip, *ndufa1*, *ndufa3*, *pcyt1a*, *ctsk*, *col6a2*, *casp2* exhibit faster evolutionary rates during long-term adaptation to deep-water environments. These genes may provide the species with advantages for survival and adaptation to environmental changes.

## Figures and Tables

**Table 1:** The sequencing information of transcriptomics.

Sample	Raw Reads	Clean Reads	Clean Reads Ratio (%)	Clean Bases (bp)	Clean Reads Q20 (%)	Clean Reads Q30 (%)
*N. bathybius*	48,063,784	47,827,824	99.51	7,144,556,370	98.01	94.45
*N. furcosus*	55,724,924	55,422,050	99.46	8,274,446,963	97.75	93.92
*N. nemurus*	52,998,374	52,690,592	99.42	7,869,665,822	97.65	93.62
*N. virgatus*	51,121,272	50,875,276	99.52	7,607,939,561	97.94	94.26
*N. aurora*	46,469,258	46,255,048	99.54	6,915,888,873	97.82	94.03
*N. japonicus*	49,153,734	48,869,976	99.42	7,311,785,453	97.13	92.37
*N. marginatus*	41,533,576	41,302,034	99.44	6,177,399,999	97.16	92.40

**Table 2:** Statistics for the assembled unigenes.

Sample	Total Number	Max Length	Min Length	Mean Length	N50	GC (%)
*N. bathybius*	32,661	33,374	201	794	1205	49.21
*N. furcosus*	37,904	37,710	201	877	1400	49.27
*N. nemurus*	38,342	60,447	201	971	1701	48.06
*N. virgatus*	41,024	43,285	201	877	1433	48.55
*N. aurora*	34,882	70,286	201	1096	1958	48.57
*N. japonicus*	40,036	67,520	201	1177	2165	47.88
*N. marginatus*	51,540	51,855	201	965	1872	47.73

**Table 3:** Numbers of unigenes annotated in the muscle tissues of seven species within the genus *Nemipterus* against four databases.

	*N. bathybius*	*N. furcosus*	*N. nemurus*	*N. virgatus*	*N. aurora*	*N. japonicus*	*N. marginatus*
NR	20,641 (63.20%)	22,907 (60.43%)	22,637 (59.04%)	25,751 (62.77%)	22,016 (63.12%)	24,115 (60.23%)	28,636 (55.56%)
KEGG	20,291 (62.13%)	22,596 (59.61%)	22,248 (58.03%)	25,356 (61.81%)	21,708 (62.23%)	23,673 (59.13%)	28,058 (54.44%)
KOG	13,648 (41.79%)	15,293 (40.35%)	14,915 (38.90%)	16,582 (40.42%)	14,802 (42.43%)	15,348 (38.34%)	18,562 (36.01%)
Swiss-Prot	16,878 (51.68%)	18,962 (50.03%)	18,567 (48.42%)	20,761 (50.61%)	18,397 (52.74%)	19,404 (48.47%)	23,225 (45.06%)
annotation genes	20,858 (63.86%)	23,178 (61.15%)	22,842 (59.57%)	25,948 (63.25%)	22,331 (64.02%)	24,154 (60.33%)	28,699 (55.68%)

Note: The percentages of unigenes annotated against each database among all unigenes are shown in parentheses.

## Data Availability

The data presented in this study are available in this article.

## Supplementary Materials

The supporting information can be downloaded at: https://www.mdpi.com/article/10.3390/ijms26157118/s1.
