# Supplementary material for: Comparative Transcriptomic Analysis for Identification of Environmental-Responsive Genes in Seven Species of Threadfin Breams (Nemipterus)
Source: Int J Mol Sci. 2025 Jul 23;26(15):7118. doi: 10.3390/ijms26157118 (PMC12346068; doi:10.3390/ijms26157118)
Supplement: Supplementary file 1 [file ijms-26-07118-s001.zip › COI Sequences.pdf]

The COI sequences used in this study

>P.caninus_seq
GAGCTGGTATGGTAGGAACCGCACTAAGCCTGCTCATTCGAGCGGAGCTTA
GCCAACCAGGCGCTCTTTTAGGAGATGACCAGATTTATAATGTTATTGTTAC
CGCTCATGCTTTTGTAATGATTTTCTTTATAGTGATGCCGATCATGATTGGAG
GATTTGGAAATTGGCTAATCCCCCTTATGATTGGGGCCCCCGATATAGCCTT
CCCCCGAATGAACAATATGAGTTTCTGGCTTCTACCACCCTCTTTCCTACTT
TTGCTCGCCTCTTCAGGAGTAGAGGCAGGGGCCGGAACAGGCTGAACTGT
GTATCCTCCCCTTGCAGGCAACCTAGCTCACGCGGGTGCATCTGTAGACCT
TACTATCTTTTCACTCCATTTAGCAGGGATCTCCTCAATTTTAGGGGCTATTA
ATTTTATTACAACTATTATTAATATGAAACCTCCTGCCATTTCACAATACCAA
ACACCCCTATTTGTATGAGCTGTCCTAATCACAGCTGTTCTTCTCCTCCTATC
CCTCCCTGTGCTTGCAGCTGGTATTACAATGCTACTAACAGATCGAAATCTA
AATACAACCTTCTTTGATCCTGCAGGAGGGGGCGACCCAATCCTCTATCAG
CACCTATTC

>N.marginatus_seq
GGGCCGGCATGGTGGGGACTGCACTAAGCCTGCTTATTCGAGCAGAGCTC
AGTCAACCAGGTTCCCTCCTAGGTGACGACCAAATTTATAACGTTATTGTTA
CGGCCCACGCTTTCGTAATAATTTTCTTCATAGTAATACCAATTATGATTGGA
GGCTTCGGAAACTGACTGATTCCTCTAATGATTGGTGCCCCTGATATGGCAT
TCCCTCGAATGAATAATATGAGCTTCTGGCTCCTGCCCCCTTCCTTCCTTCTT
CTTCTTGCCTCATCAGGCATTGAAGCAGGCGCAGGGACTGGCTGAACAGT
CTACCCCCCTCTTGCAGGCAATCTTGCTCACGCAGGAGCATCTGTCGACCT
CACCATCTTCTCTCTCCACTTAGCAGGGATTTCTTCAATTCTAGGGGCCATT
AATTTTATTACTACTATTATTAACATAAAACCTCCTGCTATGTCCCAATATCAA
ACGCCTCTCTTCGTTTGAGCCGTGCTAATTACAGCTGTTCTTCTTTTACTTTC
CCTCCCCGTACTAGCAGCCGGCATCACAATGCTTCTCACTGACCGAAACTT
AAATACAACCTTCTTTGACCCGGCAGGGGGTGGAGACCCTATTCTTTACCA
GCATCTTTTC

>N.nemurus_seq
GAGCCGGCATAGTCGGAACTGCTCTCAGCCTGTTAATTCGAGCTGAATTAA
GCCAACCAGGCGCCCTCTTAGGAGATGACCAAATCTATAATGTTATTGTAAC
AGCTCACGCTTTTGTAATAATTTTCTTTATAGTAATGCCAATTATGATTGGCG
GGTTTGGAAATTGATTAATCCCACTCATGATTGGTGCCCCTGATATGGCATTT
CCCCGTATAAATAATATAAGCTTCTGACTTCTTCCCCCCTCATTTCTTCTCCT

CCTTGCTTCTTCAGGAATTGAAGCAGGCGCAGGAACAGGTTGAACAGTCT
ACCCGCCCCTTGCAGGCAACCTAGCCCATGCAGGAGCATCTGTTGACTTAA
CCATCTTCTCCCTTCATTTGGCAGGTATTTCCTCAATCCTGGGAGCCATCAAT
TTCATTACAACCATTATTAACATGAAACCCCCTGCTATTTCCCAATATCAAAC
GCCTCTCTTCGTGTGGGCAGTCCTAATTACAGCTGTTCTCCTCCTCCTCTCT
CTTCCTGTTTTAGCAGCTGGTATTACAATACTACTAACTGACCGTAACTTAA
ATACAACTTTCTTTGATCCAGCAGGGGGCGGAGACCCCATCCTTTACCAAC
ATCTTTTC

>N.furcosus_seq
GAGCCGGCATGGTCGGAACCGCCCTAAGCCTCCTGATCCGAGCTGAACTC
AGCCAACCAGGCGCCCTCCTGGGAGACGACCAAATTTATAACGTCATCGTT
ACAGCTCACGCTTTCGTAATAATTTTCTTTATAGTAATACCAATTATGATTGG
AGGCTTCGGAAATTGATTAGTACCACTAATGATCGGCGCCCCCGATATAGCA
TTCCCCCGTATGAACAACATGAGTTTCTGACTCCTCCCCCCTTCATTCCTTC
TCCTCCTTGCCTCCTCAGGCATTGAGGCAGGTGCAGGAACAGGCTGAACA
GTTTATCCTCCCCTTGCAGGCAATCTAGCACACGCAGGGGCATCTGTAGAC
TTAACCATTTTTTCTCTTCACCTAGCAGGGATTTCTTCAATCTTAGGAGCTAT
TAACTTCATTACTACAATTATTAATATGAAACCTCCTGCTATTTCACAATATCA
AACACCCCTCTTCGTATGAGCAGTACTAATCACAGCTGTTCTCCTCCTCCTT
TCTCTTCCCGTTTTAGCGGCCGGTATTACAATGTTACTAACTGACCGAAACT
TAAACACGACTTTCTTTGACCCTGCAGGAGGGGGAGACCCCATCCTTTACC
AACATCTCTTC

>N.japonicus_seq
GGGCCGGCATAGTGGGGACTGCTCTAAGTCTGCTTATTCGGGCGGAACTTA
GCCAACCAGGTGCCCTCTTAGGTGACGATCAGATTTATAATGTTATTGTTAC
GGCTCACGCTTTCGTAATAATCTTTTTTATAGTAATACCAATTATGATCGGAG
GATTCGGAAACTGACTGGTACCACTAATGATTGGTGCCCCTGATATAGCATT
CCCTCGAATAAATAATATGAGCTTCTGACTTCTGCCCCCCTCATTCCTTCTTC
TTCTTGCTTCATCAGGCATTGAAGCAGGTGCAGGAACCGGCTGAACAGTAT
ACCCTCCCCTTGCAGGCAACCTAGCACATGCAGGAGCGTCTGTTGACCTCA
CTATTTTTTCCCTTCACTTGGCAGGGATTTCTTCAATTTTAGGGGCCATTAAT
TTTATTACAACTATTATTAATATAAAACCTCCTGCTATTTCCCAATATCAAACG
CCTCTCTTCGTCTGAGCAGTACTAATTACAGCTGTTCTTCTTCTTCTCTCCCT
TCCCGTCTTAGCAGCCGGTATTACTATACTTCTTACTGACCGAAACTTAAAC
ACAACCTTCTTTGACCCTGCAGGTGGAGGAGATCCTATTCTTTATCAACACC
TCTTC

>N.virgatus_seq
GAGCCGGCATAGTGGGGACCGCACTAAGTTTGTTAATTCGAGCAGAGCTTA
GTCAACCAGGGGCCCTCCTAGGCGACGACCAGATTTATAACGTTATTGTTAC
GGCTCACGCTTTTGTAATAATTTTCTTTATAGTAATACCAATTATGATCGGCG
GGTTCGGAAACTGACTAATCCCCCTCATGATCGGAGCCCCCGACATGGCAT
TCCCCCGAATAAATAACATAAGCTTCTGACTTTTACCCCCTTCTTTCCTTTTA
CTTCTTGCTTCGTCCGGCATTGAGGCAGGGGCAGGAACAGGCTGAACAGT
CTATCCCCCTCTTGCAGGCAACCTAGCACACGCAGGAGCATCCGTTGATTT
AACCATTTTCTCACTCCACCTGGCTGGGATTTCTTCAATTTTAGGGGCTATT
AACTTTATTACTACTATTATTAATATGAAGCCTCCAGCTATTTCCCAATACCA
AACACCCTTATTCGTATGGGCAGTTTTAATTACAGCTGTCCTCCTCCTTCTTT
CTCTTCCCGTTTTAGCAGCCGGTATTACAATGCTTCTAACTGACCGAAACCT
AAACACAACCTTCTTCGACCCTGCAGGCGGAGGAGATCCTATTCTTTACCA
ACACCTTTTC

>N.bathybius_seq
GAGCCGGCATAGTAGGAACCGCACTAAGTCTGCTTATTCGAGCTGAACTCA
GTCAACCAGGAGCCCTTTTAGGTGACGACCAAATTTATAATGTCATTGTTAC
GGCTCACGCTTTTGTAATAATTTTCTTTATAGTAATACCAATTATGATCGGCG
GGTTCGGAAACTGATTAATCCCGCTAATGATCGGGGCCCCTGATATGGCCTT
CCCTCGAATAAATAATATGAGCTTCTGGCTTTTACCCCCTTCTTTCCTTTTAC
TTCTCGCCTCATCTGGCATTGAAGCAGGGGCAGGAACAGGTTGAACAGTC
TATCCCCCTCTAGCAGGTAACCTGGCACATGCAGGGGCATCTGTTGATTTAA
CTATTTTCTCCCTTCACCTGGCTGGGATTTCTTCAATTTTAGGGGCCATCAA
CTTTATCACTACTATTTTTAATATGAAACCTCCAGCTATCTCTCAGTACCAAA
CACCCCTATTCGTTTGAGCAGTTCTTATTACAGCTGTCCTTCTCCTTCTTTCT
CTCCCCGTTTTAGCGGCCGGTATTACAATGCTTTTAACTGACCGTAATCTAA
ACACAACTTTCTTTGATCCTGCAGGCGGGGGAGATCCTATTCTTTACCAAC
ATCTTTTC

>N.aurora_seq
GGGCCGGCATAGTAGGGACCGCACTAAGTCTGCTAATCCGGGCGGAACTAA
GTCAACCAGGCGCCCTTCTAGGCGACGACCAAATTTATAATGTTATTGTTAC
GGCTCATGCTTTTGTAATAATTTTCTTTATAGTAATACCAATTATGATCGGCG
GGTTCGGAAACTGGCTAATTCCCCTTATGATCGGGGCCCCCGATATAGCATT
TCCTCGAATAAATAATATAAGCTTTTGACTTCTACCCCCTTCTTTCCTTTTAC
TTCTTGCATCCTCCGGCATTGAAGCAGGAGCAGGAACAGGTTGAACAGTC
TATCCGCCTCTTGCAGGCAACCTAGCACATGCAGGAGCATCTGTTGATTTAA

CTATTTTTTCACTTCACCTGGCCGGTATTTCTTCAATTTTAGGGGCTATTAAC
TTCATCACTACAATTATTAATATAAAACCTCCAGCCATTTCCCAATATCAAAC
ACCCCTATTCGTATGGGCAGTTCTAATTACAGCAGTTCTTCTCCTTCTTTCTC
TCCCTGTCTTAGCAGCCGGCATTACAATGCTTTTAACAGATCGAAACCTAAA
CACAACTTTTTTTGATCCTGCAGGCGGAGGAGATCCTATTCTTTACCAACAT
CTTTTC
