# Supplementary figures and images for: Comparative Transcriptomic Analysis for Identification of Environmental-Responsive Genes in Seven Species of Threadfin Breams (Nemipterus)
Source: Int J Mol Sci. 2025 Jul 23;26(15):7118. doi: 10.3390/ijms26157118 (PMC12346068; doi:10.3390/ijms26157118)

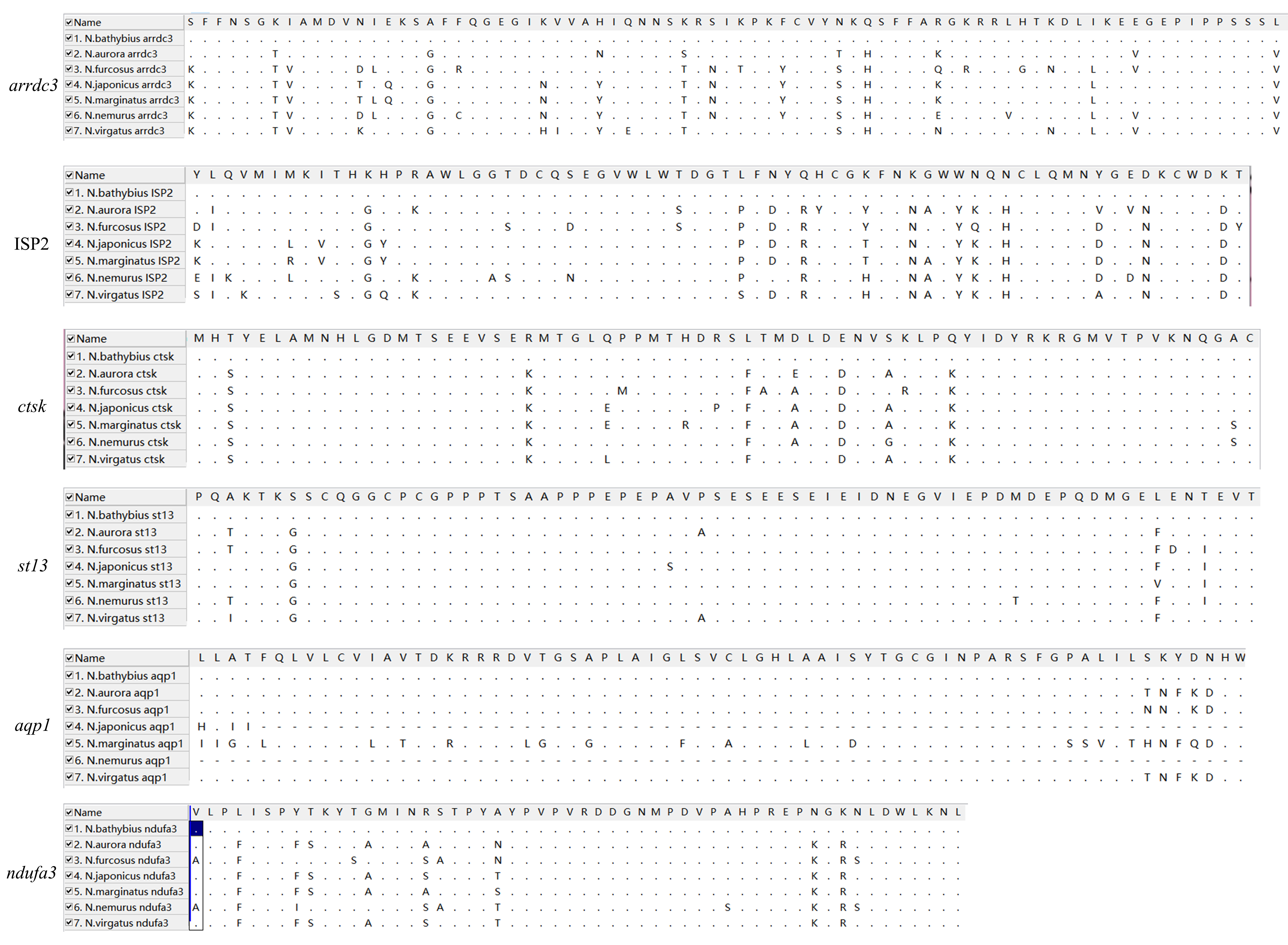

Supplement: Supplementary file 1 [file ijms-26-07118-s001.zip › Figure S2.jpg]

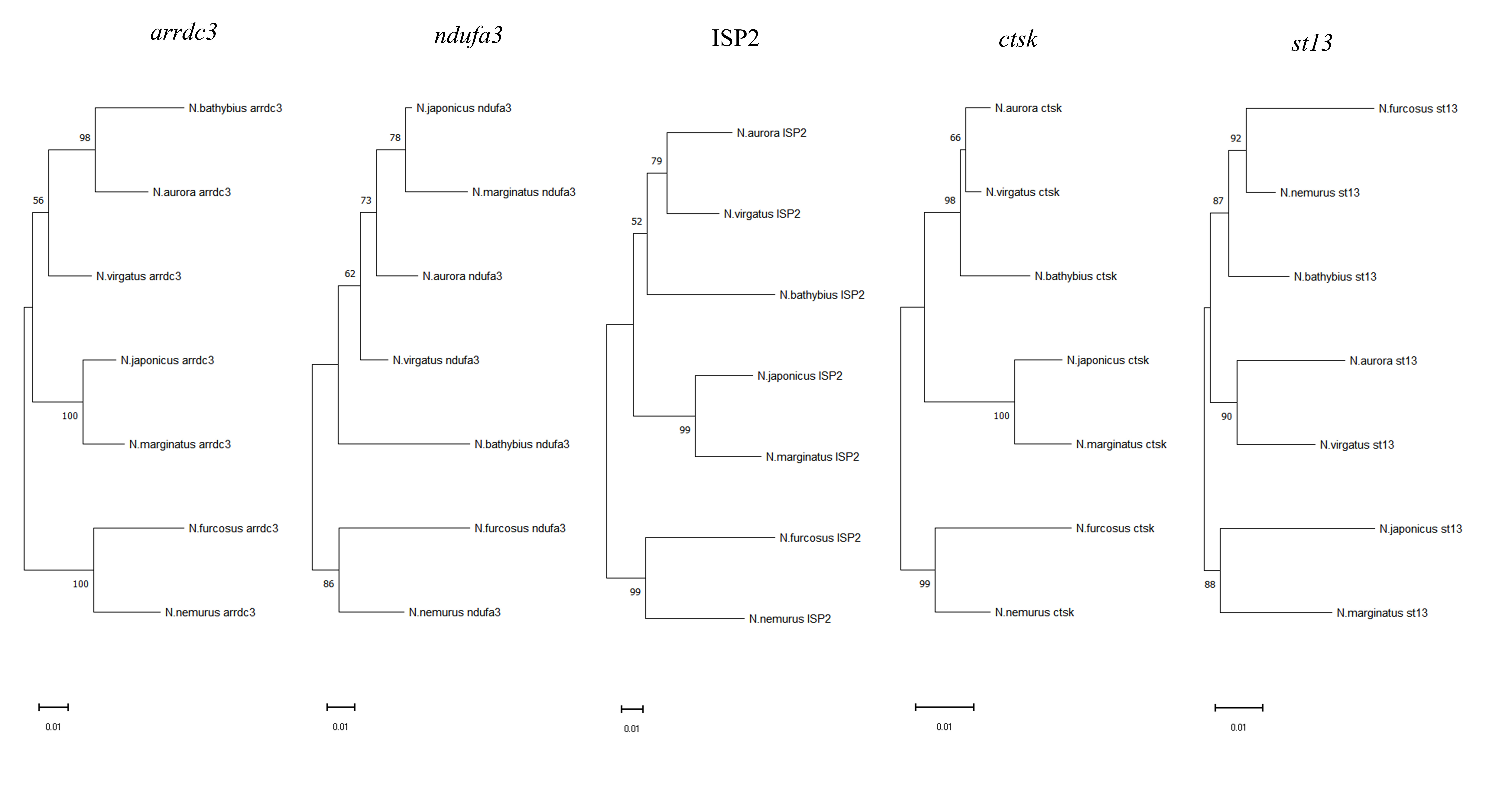

Supplement: Supplementary file 1 [file ijms-26-07118-s001.zip › Figure S1.jpg]
